# Jasmonic Acid Impairs Arabidopsis Seedling Salt Stress Tolerance Through MYC2-Mediated Repression of *CAT2* Expression

**DOI:** 10.3389/fpls.2021.730228

**Published:** 2021-10-22

**Authors:** Ru-Feng Song, Ting-Ting Li, Wen-Cheng Liu

**Affiliations:** ^1^State Key Laboratory of Crop Stress Adaptation and Improvement, State Key Laboratory of Cotton Biology, School of Life Sciences, Henan University, Kaifeng, China; ^2^Jiangsu Key Laboratory of Marine Pharmaceutical Compound Screening, Jiangsu Ocean University, Lianyungang, China

**Keywords:** salt stress, H2O2, CAT2, JA, MYC2

## Abstract

High salinity causes ionic, osmotic, and oxidative stresses to plants, and the antioxidant enzyme Catalase2 (CAT2) plays a vital role in this process, while how *CAT2* expression is regulated during plant response to high salinity remains elusive. Here, we report that phytohormone jasmonic acid (JA) impairs plant salt stress tolerance by repressing *CAT2* expression in an MYC2-dependent manner. Exogenous JA application decreased plant salt stress tolerance while the *jar1* mutant with reduced bioactive JA-Ile accumulation showed enhanced salt stress tolerance. JA enhanced salt-induced hydrogen peroxide (H_2_O_2_) accumulation, while treatment with H_2_O_2_-scavenger glutathione compromised such effects of JA on plant H_2_O_2_ accumulation and salt stress tolerance. In addition, JA repressed *CAT2* expression in salt-stressed wild-type plant but not in *myc2*, a mutant of the master transcriptional factor MYC2 in JA signaling, therefore, the *myc2* mutant exhibited increased salt stress tolerance. Further study showed that mutation of *CAT2* largely reverted lower reactive oxygen species (ROS) accumulation, higher CAT activity, and enhanced salt stress tolerance of the *myc2* mutant in *myc2 cat2-1* double mutant, revealing that *CAT2* functions downstream JA-MYC2 module in plant response to high salinity. Together, our study reveals that JA impairs Arabidopsis seedling salt stress tolerance through MYC2-mediated repression of *CAT2* expression.

## Introduction

High concentrations of sodium (Na^+^) in soil severely affects crop growth, causing enormous loss of crop quantity and quality and posing serious food security worldwide (Munns and Tester, 2008; Yang and Guo, 2018; Qin et al., 2020). Plants usually cannot avoid high salinity-induced damages by directly changing their location due to the sessile lifestyle. Plants have evolved sophisticated and complex mechanisms to respond to excess Na^+^ in the soil to survive and reproduce themselves (Munns and Tester, 2008). High salinity reduces plant water uptake and disturbs plant normal physiological processes, causing osmotic and ionic stress. The well-characterized plant Salt Overly Sensitive (SOS) pathway regulates cellular ion homeostasis by increasing Na^+^ efflux under salt stress conditions (Du et al., 2011; Zhu, 2016). In addition to ionic and osmotic stresses caused by salt stress, high salinity also elicits oxidative stress, a secondary effect, resulted from salt-induced over-accumulation of reactive oxygen species (ROS), such as hydroxyl radicals, hydrogen peroxide (H_2_O_2_), and superoxide anions (Yang and Guo, 2018). Over-accumulated ROS can damage plant biological macromolecules, such as protein, DNA, and lipids, disturbing normal physiological and metabolic processes in plant cells (Waszczak et al., 2018; Ciacka et al., 2020; Gechev and Petrov, 2020; Hasanuzzaman et al., 2020).

Antioxidant enzymes, including superoxide dismutase (SOD), catalase (CAT), peroxiredoxin, and non-enzymatic small molecules synergistically contribute to enhance plant salt stress tolerance by removing stress-induced excess ROS in plant cells (Ahmad et al., 2010; Caverzan et al., 2012). Early reports showed that mutation of *CAT2* resulted in increased H_2_O_2_ accumulation and decreased tolerance to salt stress in the Arabidopsis plant (Bueso et al., 2007). As the molecular chaperone of CAT2, protein NO CATALASE ACTIVITY1 (NCA1) facilitates the proper folding of CAT2 protein in a functional state (Li et al., 2015), thus its mutant has lower salt stress tolerance with decreased CAT activity. In addition, the mutant of a peroxisome-located small heat shock protein Hsp17.6CII is also sensitive to salt stress because Hsp17.6CII is required for the activation of CAT activity in plant response to salt stress (Wei et al., 2020). Recently, we identified Leucine Aminopeptidase2 (LAP2) as a novel CAT2-interacting protein and reported that LAP2 confers Arabidopsis plants salt stress tolerance by promoting CAT2 protein stability under high salinity (Zhang et al., 2021). These reports reveal that CAT indeed plays an important role in plant salt stress tolerance. To respond to salt stress, plants also activate *CAT* expression to increase antioxidant activity, alleviating salt-induced oxidative damages to plant cells (Xing et al., 2007; Munns and Tester, 2008; Yang and Guo, 2018). However, transcriptional factors regulating *CAT2* expression in plant response to salt stress have not been reported so far, and the regulatory mechanism underlying *CAT2* expression remains to be further elucidated.

Phytohormones play important but different roles in plant response and tolerance to salt stress. Roles of jasmonic acid (JA) in plant growth, development, and environmental responses, such as floral development, senescence, fruit ripening, pathogen infection, insect attack, wounding, and cold, have been widely documented (Gfeller et al., 2010; Santino et al., 2013; Yuan et al., 2017; Schluttenhofer, 2020), while its function in plant salt stress tolerance remains elusive and even controversial. Early reports showed that exogenous application of JA or methyl JA (MeJA) confers increased salt stress tolerance in pepper, rice, *Pisum sativum*, and barley (Moons et al., 1997; Tsonev et al., 1998; Velitchkova and Fedina, 1998). A similar conclusion was later verified in other plant species, including wheat and soybean (Ji et al., 2009; Qiu et al., 2014). These reports reveal a positive role of JA in plant salt stress tolerance. However, some studies have reached the opposite conclusion that JA negatively impacts plant salt stress tolerance. For example, a rice mutant of the JA biosynthetic enzyme ALLENE OXIDE CYCLASE (AOC) has a lower JA accumulation but higher salt stress tolerance (Hazman et al., 2015). In addition, overexpression of *CYP94*, a gene encoding an enzyme-inactivating bioactive form of JA, JA-Ile, enhances the viability of rice plants under saline conditions (Kurotani et al., 2015). These studies documented the involvement of JA in plant salt stress response, however, the mechanism underlying JA-regulated plant salt stress tolerance remains to be further elucidated.

As the master transcription factors in JA signaling pathway, MYC2, MYC3, and MYC4 function redundantly in diverse JA-dependent functions and various plant environmental responses (Zhang et al., 2020; Li et al., 2021; Wang et al., 2021). A previous study documented that mutants of *MAPK KINASE2* (*MKK2*) and *MKK3* have increased *MYC2* expression, enhanced JA sensitivity but decreased seed germination rate under saline conditions, suggesting the involvement of JA signaling component MYC2 in plant salt stress response (Takahashi et al., 2007). However, their roles in plant salt stress tolerance remain largely unknown. Here, we report that JA negatively regulates plant salt stress tolerance by repressing *CAT2* expression in an MYC2-dependent manner. Exogenous JA treatment reduces plant salt stress tolerance by increasing high salinity-induced H_2_O_2_ accumulation, while ROS scavenger glutathione (GSH) compromises such effects of JA. The expression and enzymatic activity of CAT2 are repressed by JA in the wild-type plant but not in the *myc2* mutant, and thus the *myc2* mutant was more tolerant to salt stress than the wild-type plant in the presence of JA. Furthermore, mutation of *CAT2* largely reverts the salt stress tolerance phenotype of the *myc2* mutant in *myc2 cat2-1* double mutant. Together, our study reveals that JA impairs Arabidopsis seedling salt stress tolerance through MYC2-mediated repression of *CAT2* expression.

## Materials and Methods

### Plant Material and Growth Conditions

Arabidopsis (*Arabidopsis thaliana*) ecotype Columbia was used in this study. The β-glucuronidase (GUS) reporter line *CAT2pro::GUS* and mutants *myc2* (SALK_017005), *cat2-1* (SALK_076998), and *myc2 cat2-1* were previously reported (Zhang et al., 2020). The mutant *jar1* (CS8072) was obtained from the Arabidopsis Biological Resource Center (ABRC). Seeds were surface sterilized for 5 min in 5% commercial bleach, washed three times with sterile water, and plated on 1/2 Murashige and Skoog (MS) medium (pH 5.8) (Sigma-Aldrich, St. Louis, MO, USA) containing 1% sucrose and 1% agar. Plant seeds were stratified at 4°C for 3 days in the dark and then transferred to chambers. The seedlings grown vertically at 22°C and 100 μmol m^−2^ s^−1^ illumination under 16 h light/8 h dark conditions for 5 days were transferred to 1/2 MS medium without or with NaCl or MeJA for another 5 days, and the lengths of newly grown roots and seedling fresh weight were measured and statistically analyzed.

### 3,3-Diaminobenzidine (DAB) Staining

The treated or untreated seedlings were used for DAB to assay H_2_O_2_ accumulation as we described previously (Zhang et al., 2020a,b). Briefly, the seedlings were incubated in freshly prepared DAB staining solution (1 mg/ml DAB and 0.1% Tween 20 in 10 mM Na_2_HPO_4_) for about 8 h and then rinsed with 70% ethanol several times to remove the chlorophyll. The images of the leaves were captured using a digital camera.

### Detection of CAT Activity

The salt- or MeJA-treated or untreated seedlings were ground to a fine powder under liquid nitrogen and suspended in freshly prepared cold protein extraction buffer (50 mM potassium phosphate buffer, pH 7.8, 0.2 mM ethylenediaminetetraacetic acid (EDTA)-Na_2_, 0.1 mM ascorbic acid, and 1% polyvinyl polypyrrolidone, PVPP). After centrifugation at 12,000 *g* for 10 min at 4°C, the supernatant was transferred to a new tube for further use. The protein concentration was assayed by the Bradford method, and CAT activity was determined according to the published methods (Aebi, 1984). CAT activity was assayed by monitoring the consumption of H_2_O_2_ at 240 nm.

### Quantitative Real-Time PCR (qRT-PCR)

Treated or untreated seedlings were collected for total RNA isolation, first-strand cDNA synthesis, and qRT-PCR as we described previously (Liu et al., 2017a; Zhang et al., 2020a). The constitutively expressed *ACTIN2/8* gene was used as an internal control. All experiments were repeated at least three times. The primer sequences are listed in Supplementary Table 1.

### GUS Staining

The GUS histochemical staining experiment was performed according to our previously reported method (Zhang et al., 2020a,b). About 5-day-old *CAT2::GUS* transgenic seedlings were treated with 0.25 μM MeJA or 125 mM NaCl for 1 day and then incubated at 37°C in GUS staining solution (100 mM sodium phosphate buffer, pH 7.5, 10 mM EDTA, 0.5 mM potassium ferricyanide, 0.5 mM potassium ferrocyanide, 1 mM 5-bromochloro-3-indolyl-b-D-glucuronide, and 0.1 % Triton X-100). Then, the seedlings were rinsed with 70% ethanol several times to remove the chlorophyll, and images were captured using a digital camera.

### Measurement of JA Levels

Jasmonic acid levels were determined according to our previously reported methods (Yuan et al., 2017; Zhang et al., 2020a). Briefly, about 200 mg of the treated and untreated seedlings were collected and ground into fine powder under liquid nitrogen. Dihydrojasmonic acid (DHJA) of 20 ng was added as an internal standard for JA. The purified samples were then methylated by diazomethane, re-suspended in 100 μl ethyl acetate, and analyzed by gas chromatography-mass spectrometry-selected ion monitoring (GC/MS-SIM) (Trace GC Ultra/ISQ, Thermo Fisher Scientific, Waltham, MA, USA). The compounds were separated on an Rtx-5MS (30 mm × 0.25 mm × 0.25 mm) column. The chromatographic parameters were as follows: 40°C for 1 min after injection, followed by sequential temperature ramps of 25°C/min to 150°C, 5°C/min to 200°C, 10°C/min ramp to 240°C, and 240°C for 10 min. The monitored ions were m/z 151 and 153 for JA and DHJA, respectively.

### Statistical Analysis

Data are means (±SD) of three biological replicates, and the asterisks indicate statistically significant differences (^*^p < 0.05, ^**^p < 0.01, and ^***^p < 0.001, Student's *t*-test). Bars with different letters indicate significant differences at *p* < 0.05 by two-way ANOVA with Tukey's multiple comparison test.

## Results

### JA Reduces Plant Salt Stress Tolerance

**Figure 1:**
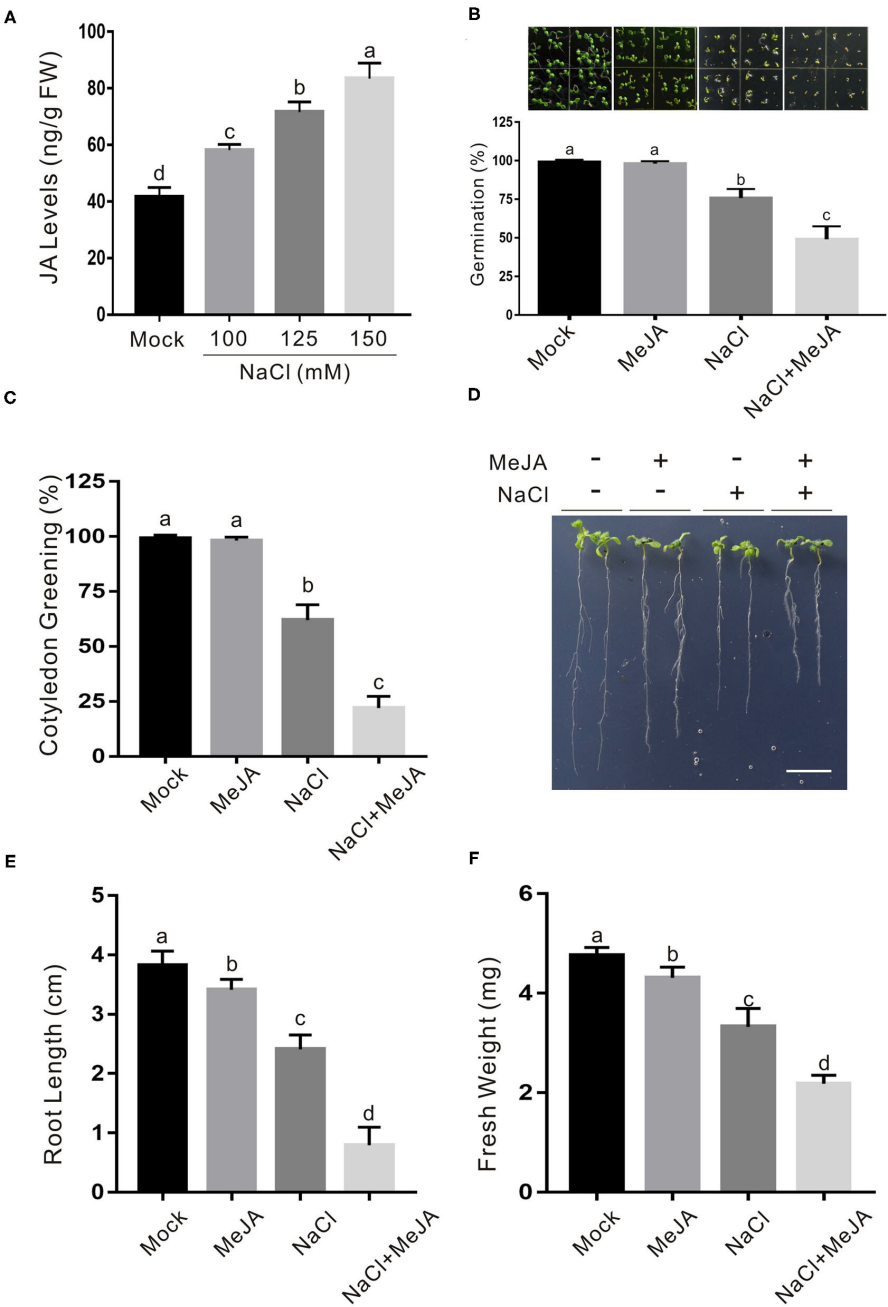
JA increases plant sensitivity to salt stress. **(A)** JA levels in salt-treated or untreated wild-type plant seedlings. The 5-day-old wild-type plant seedlings were treated with 0, 100, 125, or 150 mM NaCl for 5 days, and used for the determination of JA levels. Data are means (±SD) of three biological replicates. Bars with different letters indicate significant differences at *p* < 0.05, revealed using a one-way ANOVA with a Tukey's multiple comparison test. **(B)** Images (top) and percentages of seed germination (bottom) of 5-day-old wild-type plant seedlings treated with or without 2.5 μM MeJA or 125 mM NaCl. **(C)** Percentages of green cotyledons of 5-day-old wild-type plants treated with 2.5 μM MeJA and/or 125 mM NaCl. **(D)** Images of NaCl- and/or MeJA-treated wild-type plant seedlings. The 5-day-old wild-type plant seedlings were treated with 2.5 μM MeJA and/or 125 mM NaCl for 5 days. Bar = 1.0 cm. **(E, F)** The newly grown root length **(E)** and seedling fresh weight **(F)** of NaCl- and/or MeJA-treated wild-type plant seedlings. The 5-day-old wild-type plant seedlings were treated with 2.5 μM MeJA and/or 125 mM NaCl for 5 days, and then the newly grown root length and seedling fresh weight were determined. Data are means (±SD) of three biological replicates. Bars with different letters indicate significant differences at *p* < 0.05, revealed using a two-way ANOVA with a Tukey's multiple comparison test. JA, jasmonic acid; MeJA, methyl JA.

Previous reports documented different roles of JA in salt stress tolerance in different plant species (Velitchkova and Fedina, 1998; Qiu et al., 2014; Hazman et al., 2015; Kurotani et al., 2015; Valenzuela et al., 2016). Our recent report showed that JA represses *CAT2* expression to stimulate leaf senescence (Zhang et al., 2020a), shedding a light on whether and how JA affect plant salt stress response through the modulation of *CAT2* expression. To understand how JA involves plant response to high salinity, we first determined JA accumulation treated with or without salt stress. About 5-day-old wild-type plant seedlings were transferred onto 1/2 MS medium containing different concentrations of additional NaCl and treated for 5 days. Our results showed that JA accumulation in salt-treated plants was significantly higher than that in the untreated control (Figure 1A), indicating that salt stress induces JA accumulation in plants.

To dissect the effect of JA on salt stress response in Arabidopsis, we examined the seed germination of the wild-type plant on 1/2 MS medium with the high salinity in the presence or absence of JA. Our results showed that low concentration of exogenous JA alone did not but high salinity significantly affected seed germination and cotyledon greening process, and exogenous JA further enhanced the salt stress effects (Figures 1B,C), suggesting a negative regulatory role of JA in plant salt stress tolerance. To verify this, 5-day-old wild-type plant seedlings were subjected to high salinity in the presence or absence of JA. Similarly, JA also increased plant sensitivity to salt stress as evidenced by shorter newly grown roots and lower seedling fresh weight in salt plus JA-treated plants compared with those of the salt-treated plants (Figures 1D–F). Consistently, two-way ANOVA analysis showed that the interaction between the two treatments (MeJA and NaCl) in Figure 1F had significant differences (Supplementary Table 2), further supporting that exogenous JA decreases plant seedling salt stress tolerance.

**Figure 2:**
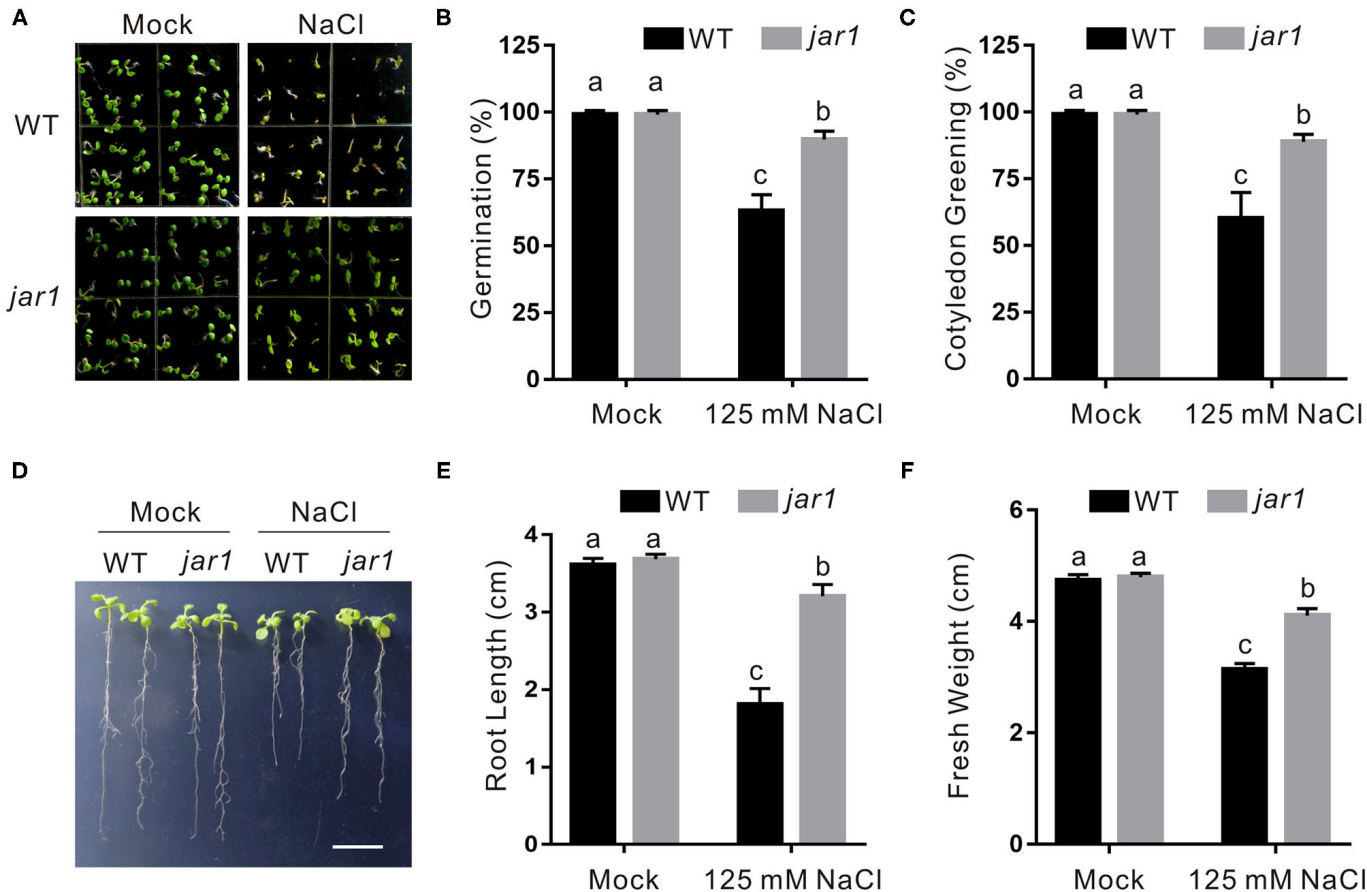
The *jar1* mutant is insensitive to salt stress. **(A)** Images of 5-day-old wild-type and *jar1* mutant plants treated with or without 125 mM NaCl. **(B, C)** Percentages of seed germination **(B)** and green cotyledons **(C)** of 5-day-old wild-type and *jar1* mutant plant treated with or without 125 mM NaCl. **(D)** Images of wild-type and *jar1* mutant seedlings were treated with or without high salinity. The 5-day-old wild-type and *jar1* mutant plant seedlings were treated with 0 or 125 mM NaCl for 5 days. Bar = 1.0 cm. **(E, F)** The newly grown root length **(E)** and seedling fresh weight **(F)** of wild-type and *jar1* mutant seedlings were treated with or without high salinity. The 5-day-old wild-type and *jar1* mutant plant seedlings were treated with 0 or 125 mM NaCl for 5 days, and then the newly grown root length and seedling fresh weight were determined. Data are means (±SD) of three biological replicates. Bars with different letters indicate significant differences at *p* < 0.05, revealed using a one-way ANOVA with a Tukey's multiple comparison test.

To confirm the role of endogenous JA in plant salt stress tolerance, a loss-of-function mutant of *JASMONATE RESISTANT1* (*JAR1*), *jar1*, which is defective in catalyzing the formation of bioactive JA, JA-Ile (Staswick et al., 2002), was employed. Compared with the wild-type plant, the *jar1* mutant had higher germination and cotyledon greening rates when subjected to salt stress (Figures 2A–C). Consistently, the *jar1* mutant exhibited higher salt stress tolerance than the wild-type plant in terms of newly grown root length and seedling fresh weight (Figures 2D–F), further supporting that JA represses plant tolerance to salt stress.

### JA Promotes Salt-Induced H_2_O_2_ Accumulation

**Figure 3:**
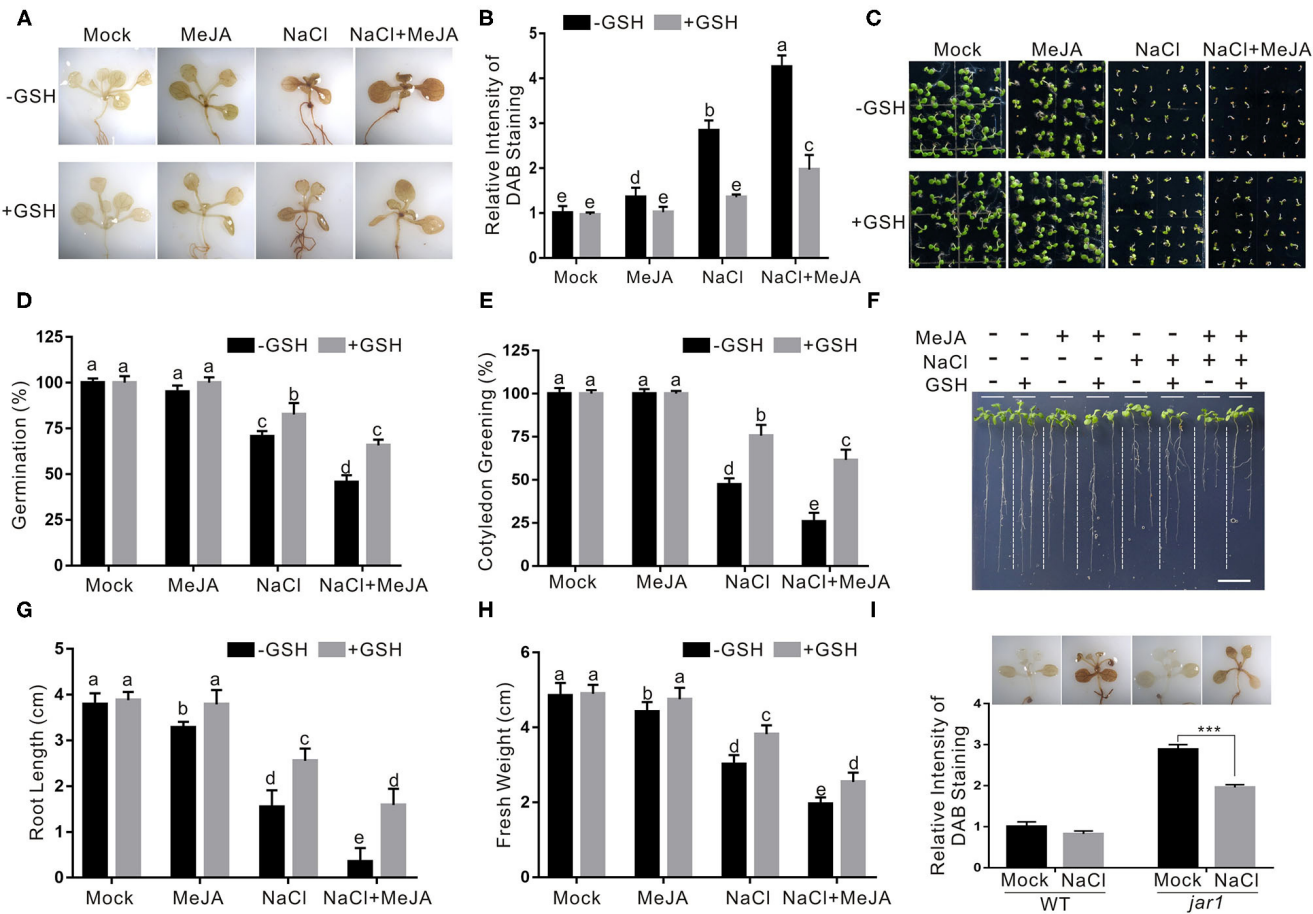
JA increases plant salt stress sensitivity by promoting ROS accumulation. **(A)** The DAB staining images of leaves from the wild-type plants treated with MeJA or NaCl in the presence or absence of GSH. The 5-day-old wild-type seedlings were treated with 2.5 μM MeJA and/or 125 mM NaCl in the presence or absence of 500 μM GSH for 5 days and used for DAB staining. **(B)** The relative DAB staining intensity in (A). The DAB staining intensity of the untreated wild-type leaves without treatment was set to 1. **(C)** Images of 5-day-old wild-type plants treated with or without 2.5 μM MeJA or 125 mM NaCl in the presence or absence of GSH. **(D, E)** Percentages of seed germination **(D)** and green cotyledons **(E)** of 5-day-old wild-type plant treated with or without 2.5 μM MeJA or 125 mM NaCl in the presence or absence of GSH. **(F)** Images of wild-type plant seedlings treated with MeJA or NaCl in the presence or absence of GSH. The 5-day-old wild-type plant seedlings were treated with 2.5 μM MeJA and/or 125 mM NaCl in the presence or absence of 500 μM GSH for 5 days. Bar = 1.0 cm. **(G, H)** The newly grown root length **(G)** and seedling fresh weight **(H)** of wild-type plant seedlings treated with MeJA or NaCl in the presence or absence of GSH. The 5-day-old wild-type plant seedlings were treated with 2.5 μM MeJA and/or 125 mM NaCl in the presence or absence of 500 μM GSH for 5 days, and then the newly grown root length and seedling fresh weight were determined. **(I)** The DAB staining images (top) and the relative DAB staining intensity (bottom) of leaves from the wild-type and *jar1* mutant plants treated with NaCl. The 5-day-old wild-type seedlings were treated with 125 mM NaCl for 5 days and used for DAB staining. The DAB staining intensity of the untreated wild-type leaves without treatment was set to 1. Data are means (±SD) of three biological replicates. Asterisks indicate significant differences using a Student's *t*-test (***p < 0.001). Bars with different letters indicate significant differences at *p* < 0.05, revealed using a one-way ANOVA with a Tukey's multiple comparison test. MeJA, methyl JA; ROS, reactive oxygen species; DAB, 3,3-diaminobenzidine; GSH, scavenger glutathione.

Salt stress severely disturbs normal cellular physiological processes in plants, resulting in extensively higher accumulation of ROS, such as H_2_O_2_. It has been reported that increased antioxidant ability confers plants an enhanced salt stress tolerance (Hernandez et al., 2000). Therefore, we sought to investigate whether JA reduces plant salt stress tolerance through changing H_2_O_2_ accumulation by performing a DAB staining experiment. Our results showed that the wild-type plants treated with both salt stress and JA had obviously higher H_2_O_2_ accumulation than the salt-stressed plants (Figures 3A,B), indicating that exogenous JA promotes salt stress-induced H_2_O_2_ accumulation.

To test whether the effect of JA on plant salt stress tolerance was primarily due to its promotion of H_2_O_2_ accumulation, a ROS GSH was used to treat the plant in the presence of high salinity and JA. Our results showed that exogenously applied GSH significantly reduced the H_2_O_2_ accumulation of the plants treated with JA and salt stress (Figures 3A,B). In addition, seed germination, root length, and fresh weight were higher in both salt- and GSH-treated plants than those in the salt-treated control (Figures 3C–H). We also detected H_2_O_2_ levels in the *jar1* mutant treated with salt stress. We found that, consistent with its increased salt stress tolerance, the *jar1* mutant accumulates less H_2_O_2_ than the wild-type plant when treated with high salinity (Figure 3I). These results indicate that increased JA impairs plant salt stress tolerance at least partially due to promoting ROS accumulation.

### JA Represses CAT2 Expression Through MYC2 in Plant Response to Salt Stress

**Figure 4:**
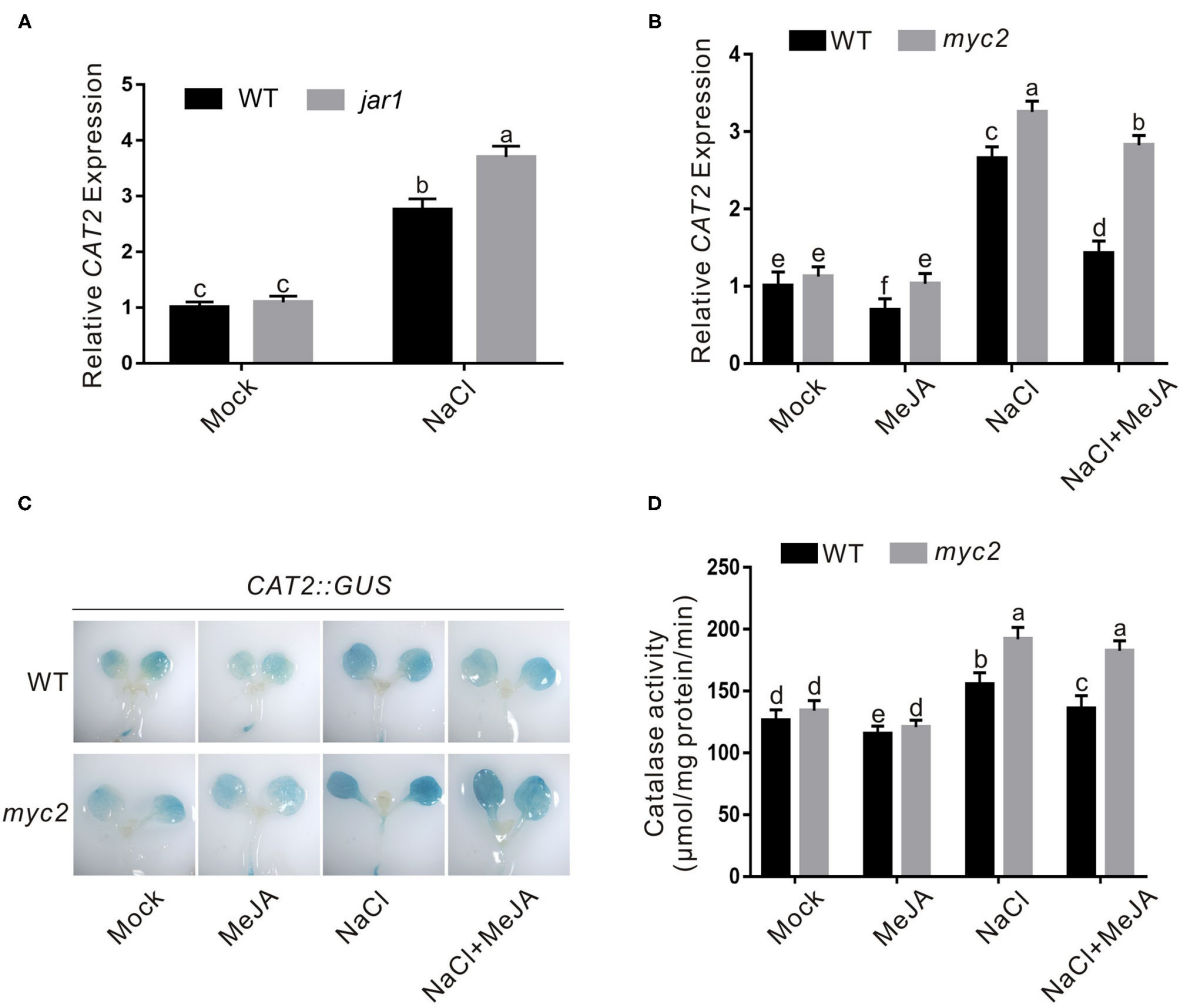
JA represses salt-induced *CAT2* expression. **(A)** The expression of *CAT2* in the wild-type and *jar1* mutant seedlings treated with NaCl. The 5-day-old wild-type and *jar1* mutant seedlings treated with or without 125 mM NaCl for 1 day were used to assay *CAT2* expression by qRT-PCR. *ACTIN2/8* was used as the reference gene. **(B)** The expression of *CAT2* in the wild-type and *myc2* mutant seedlings treated with MeJA and/or NaCl. The 5-day-old wild-type and *myc2* mutant seedlings treated with or without 2.5 μM MeJA and/or 125 mM NaCl for 1 day were used to assay *CAT2* expression by qPCR. *ACTIN2/8* was used as the reference gene. **(C)** The GUS staining images of *CAT2::GUS* and *myc2 CAT2::GUS* plants treated with MeJA and/or NaCl. The 5-day-old *CAT2::GUS* and *myc2 CAT2::GUS* plant seedlings were treated with or without 2.5 μM MeJA and/or 125 mM NaCl for 1 day, and used for GUS staining. **(D)** Catalase activity in the wild-type and *myc2* mutant seedlings treated with MeJA and/or NaCl. The 5-day-old wild-type and *myc2* mutant seedlings treated with or without 2.5 μM MeJA and/or 125 mM NaCl for 1 day. Data are means (±SD) of three biological replicates. Bars with different letters indicate significant differences at *p* < 0.05, revealed using a one-way ANOVA with a Tukey's multiple comparison test. MeJA, methyl JA; GUS, β-glucuronidase; CAT2, Catalase2; qRT-PCR, quantitative real-time PCR.

Our above results showed that JA weakens plant salt stress tolerance through increasing H_2_O_2_ accumulation, prompting us to further study whether CAT is regulated by JA in plant response to salt stress as JA downregulates *CAT2* expression during leaf senescence in our previous report (Zhang et al., 2020a). To do this, we first assayed the expression of *CAT2* in the wild-type and *jar1* mutant seedlings treated with high salinity, and our qRT-PCR results showed that salt stress treatment extensively induced the expression of *CAT2* both in the wild-type and *jar1* mutant, while the *jar1* mutant had significantly higher *CAT2* expression than the wild-type (Figure 4A), revealing that JA negatively regulates *CAT2* expression in plant under salt stress conditions. To strengthen this note, we also examined *CAT2* expression in salt-treated wild-type plant seedlings in the presence or absence of JA. We found that high salinity stress activates *CAT2* expression while JA dampened its expression in the wild-type plant (Figure 4B). Such effect of JA on *CAT2* expression in plant response to salt stress was confirmed by using the transgenic plant of *GUS* reporter gene driven by *CAT2* promoter (Zhang et al., 2020a), and our results showed that the GUS staining in salt-treated *CAT2::GUS* plants was darker than that in the untreated control while this staining was extensively repressed by JA (Figure 4C), indicating that JA downregulates *CAT2* expression in plant response to high salinity. In line with this, the CAT activity in both JA and salt-treated wild-type plants was significantly lower than that of the salt-treated plants (Figure 4D).

MYC2 is a master transcriptional factor regulating the expression of most JA downstream genes (Kazan and Manners, 2013; Santino et al., 2013). Therefore, we assessed whether MYC2 involves JA-regulated *CAT2* expression in plant response to salt stress. We treated the wild-type and *myc2* mutant with the high salinity in the presence or absence of JA and assayed their *CAT2* expression using qRT-PCR. Our results showed that salt-induced *CAT2* expression was largely repressed by JA in the wild-type plant but not in the *myc2* mutant (Figure 4B). In addition, the GUS staining in the *myc2 CAT2::GUS* plant was darker than that in the *CAT2::GUS* plant when treated with both salt and JA (Figure 4C), revealing that MYC2 is required for JA-mediated suppression of *CAT2* expression in plant response to salt stress. Further, we examined the CAT activity in the wild-type and *myc2* mutant plants treated with salt and JA, and we found that JA treatment inhibited CAT activity in salt-treated wild-type plant while such effect of JA was largely compromised in the *myc2* mutant (Figure 4D), further supporting the role of MYC2 in JA-mediated repression of *CAT2* expression in plant response to salt stress.

**Figure 5:**
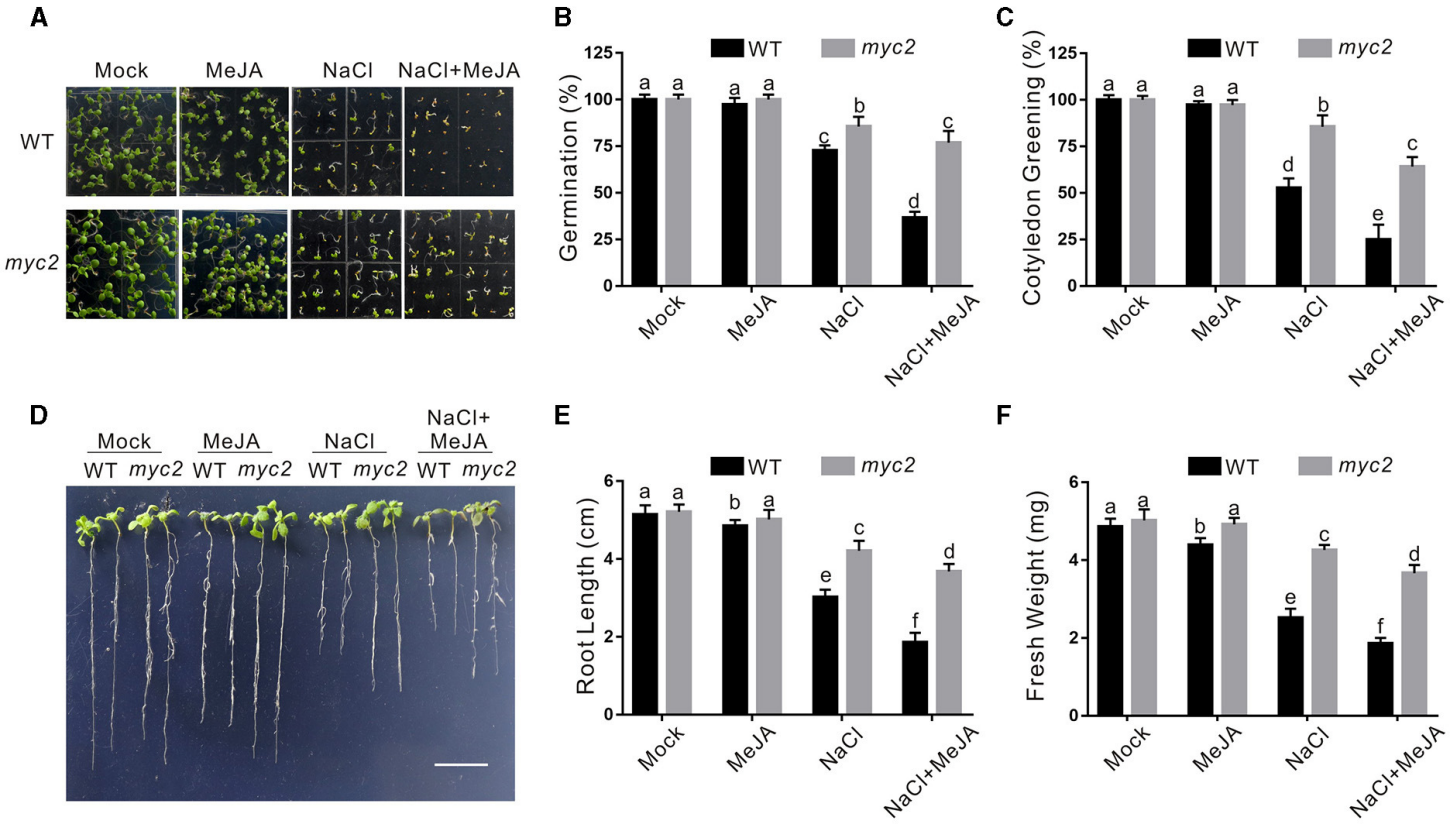
The *myc2* mutant is insensitive to salt stress. **(A)** Images of 5-day-old wild-type and *myc2* mutant plants treated with or without 2.5 μM MeJA and/or 125 mM NaCl. **(B, C)** Percentages of seed germination **(B)** and green cotyledons **(C)** of 5-day-old wild-type plants treated with or without 2.5 μM MeJA or 125 mM NaCl. **(D)** Images of wild-type and *myc2* mutant plant seedlings treated with MeJA or NaCl. The 5-day-old wild-type and *myc2* mutant plant seedlings were treated with or without 2.5 μM MeJA and/or 125 mM NaCl for 5 days. Bar = 1.0 cm. **(E, F)** The newly grown root length **(E)** and seedling fresh weight **(F)** of wild-type and *myc2* mutant plant seedlings treated with MeJA or NaCl. The 5-day-old wild-type and *myc2* mutant plant seedlings were treated with or without 2.5 μM MeJA and/or 125 mM NaCl for 5 days, and then the newly grown root length and seedling fresh weight were determined. Data are means (±SD) of three biological replicates. Bars with different letters indicate significant differences at *p* < 0.05, revealed using a one-way ANOVA with a Tukey's multiple comparison test. MeJA, methyl JA.

Increased *CAT2* expression and CAT activity of the *myc2* mutant suggested higher salt stress tolerance. Thus, we evaluated the tolerance of the *myc2* mutant to high salinity. Our results showed that the *myc2* mutant seeds had higher germination and cotyledon greening rate than the wild-type plant when germinating on 1/2 MS medium containing high salinity plus or minus JA (Figures 5A–C). In addition, the *myc2* mutant exhibited higher salt stress tolerance than the wild-type plant when challenged with salt stress as evidence by longer newly grown root and higher seedling fresh weight of the mutant in the presence or absence of JA (Figures 5D–F). These results clearly indicated that MYC2 participates in JA-mediated negative regulation of plant salt stress tolerance.

### CAT2 Functions Downstream of JA-MYC2 Module in Plant Salt Stress Tolerance

**Figure 6:**
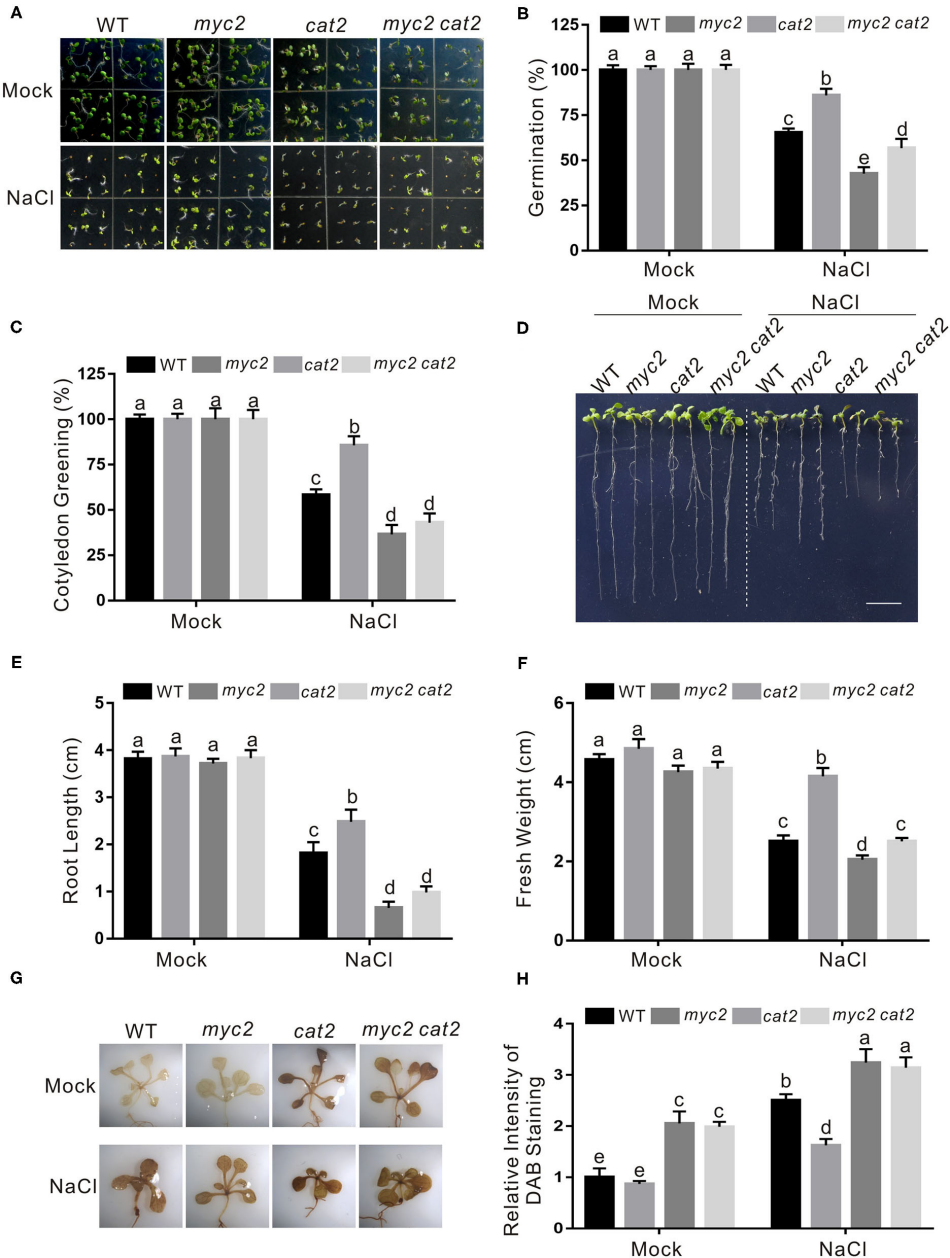
*CAT2* mutation reverts the insensitivity phenotype of *myc2* to salt stress. **(A)** Images of 5-day-old wild-type, *myc2, cat2-1, and myc2 cat2-1* mutant plants treated with or without 125 mM NaCl. **(B, C)** Percentages of seed germination **(B)** and green cotyledons **(C)** of 5-day-old wild-type, *myc2, cat2-1*, and *myc2 cat2-1* mutant plant treated with or without 125 mM NaCl. **(D)** Images of wild-type, *myc2, cat2-1*, and *myc2 cat2-1* mutant plant seedlings treated with NaCl. The 5-day-old wild-type, *myc2, cat2-1, and myc2 cat2-1* mutant plant seedlings were treated with or without 125 mM NaCl for 5 days. Bar = 1.0 cm. **(E, F)** The newly grown root length **(E)** and seedling fresh weight **(F)** of wild-type, *myc2, cat2-1*, and *myc2 cat2-1* mutant plant seedlings treated with NaCl. The 5-day-old wild-type, *myc2, cat2-1, and myc2 cat2-1* mutant plant seedlings were treated with or without 125 mM NaCl for 5 days, and then the newly grown root length and seedling fresh weight were determined. **(G)** The DAB staining images of leaves from the wild-type, *myc2, cat2-1*, and *myc2 cat2-1* mutant plant seedlings treated with NaCl. The 5-day-old wild-type, *myc2, cat2-1*, and *myc2 cat2-1* mutant plant seedlings treated with or without 125 mM NaCl for 5 days and used for DAB staining. **(H)** The relative DAB staining intensity in **(G)**. The DAB staining intensity of the untreated wild-type leaves without treatment was set to 1. Data are means (±SD) of three biological replicates. Bars with different letters indicate significant differences at *p* < 0.05, revealed using a one-way ANOVA with a Tukey's multiple comparison test. Cat2, Catalase2; DAB, 3,3-diaminobenzidine.

Our above results showed that JA suppresses *CAT2* expression in an MYC2-dependent manner in plant response to salt stress. To genetically support this note, we further determined the salt stress phenotype of the *myc2 cat2-1* double mutant. We found that the *cat2-1* mutant was indeed very sensitive to high salinity as previously reported (Bueso et al., 2007; Li et al., 2015), while the increased salt stress tolerance of the *myc2* mutant was completely reverted by the mutation of *CAT2* in *myc2 cat2-1* mutant in terms of seed germination, newly grown root length, and seedling fresh weight (Figures 6A–F). Consistently, lower ROS accumulation in the *myc2* mutant compared with that in the wild-type plant was significantly increased by the *cat2-1* mutation (Figures 6G,H).

Together with the above results, our study reveals that JA reduces Arabidopsis plant salt stress tolerance by repressing *CAT2* expression in an MYC2-dependent manner.

## Discussion

Jasmonic acid, as a major defense phytohormone, plays a key role in plant resistance to insects and pathogens especially necrotrophs by activating a large number of defense-related genes (Santino et al., 2013; Wasternack and Song, 2017). In our study, we showed that exogenous JA significantly represses plant tolerance to salt stress at least by affecting antioxidant ability through downregulating *CAT2* expression in Arabidopsis. However, we also speculated that JA may also impair plant salt stress tolerance by disturbing the balance of plant growth, biotic stress resistance, and abiotic stress tolerance as JA activates defense-related genes and thus interfering with plant growth and stress response under saline conditions. In our previous report, we showed the negative regulatory role of MYC2 in *CAT2* expression during JA-induced leaf senescence (Zhang et al., 2020a), which is consistent with our results that MYC2 represses *CAT2* expression in JA-modulated plant salt stress tolerance. Considering that MYC2 can bind *CAT2* promoter *in vitro* and *in vivo* as we previously reported (Zhang et al., 2020a), we speculate that MYC2 also binds to the *CAT2* promoter and repress its expression to dampen plant salt stress tolerance.

The JASMONATE-ZIM-DOMAIN (JAZ) proteins interact with repress JA downstream transcriptional factors, such as MYC2, thus, functioning as repressors of JA signaling. It is reported that repression of *OsJAZ9* in rice confers the plant increased salt stress tolerance while overexpression of these genes result in higher sensitivity to the stress (Wua et al., 2015), which supports a study that activated JA signaling by JA represses plant salt stress tolerance.

Catalase is a major antioxidase that primarily catalyzes the decomposition of H_2_O_2_, thus playing a vital role in plant diverse stress responses. In addition to antioxidases, plants also have multiple enzymes catalyzing the formation of ROS, such as NADPH oxidase, Glycolate Oxidase (GOX), and Acyl-CoA Oxidase (ACX) (Mittler, 2017; Liu et al., 2017b). To investigate whether these ROS-producing enzymes have roles in salt- or JA-regulated H_2_O_2_ accumulation, we examined their expression in the plant seedlings treated with salt stress in the presence or absence of JA. Our qRT-PCR results showed that high salinity treatment activated the expression of *RBOHD, RDOHF*, and *ACX2*, but not *GOX1* or *ACX3*, while JA upregulated the expression of *RBOHD* and *RDOHF* but downregulated the expression of *GOX1, ACX2*, and *ACX3* (Supplementary Figure 1). When both salt stress and JA were employed to treat the seedlings, the expression of *RBOHD* and *RBOHF* was significantly induced while the expression of *GOX1* and *ACX3* was suppressed compared to that in the untreated control plants (Supplementary Figure 1). These data suggested that RBOHD and RBOHF maybe also involved in salt- or JA-induced H_2_O_2_ accumulation by promoting ROS production. This hypothesis is supported by the previous report that both RBOHD and RBOHF are necessary for MeJA-induced stomatal closure by promoting ROS production in guard cells (Suhita et al., 2004). Extensive experiments are required to explore whether and how RBOHD and RBOHF function in JA-regulated plant salt stress response.

Recently, a report documented that MYC2 imparts salt stress tolerance by downregulating the expression of *delta1-pyrroline-5-carboxylate synthase1* (*P5CS1*) that encodes a rate-limiting enzyme for proline synthesis (Verma et al., 2020). This study suggests the negative effect of JA on plant salt stress tolerance, while whether and how JA affects plant salt stress response through MYC2 remains elusive. Together with this report and our study, we speculate that JA elicits its negative regulatory role in plant salt stress tolerance by repressing the expression of *CAT2* and *P5CS1*, leading to decreased antioxidant and osmotic-adjustment abilities in an MYC2-dependent manner. In addition to oxidative and osmotic stress damages, high salinity also causes ionic stress to plant (Julkowska and Testerink, 2015; Ryu and Cho, 2015). Whether and how JA has a role in the SOS pathway which is related to the efflux of excess Na^+^ remains unclear and is worthy to be experimentally explored.

It is noteworthy that JA and the components in its signaling pathway elicit positive effects on plant salt stress tolerance in addition to their negative roles during plant response to salt stress. For example, preliminary JA application confers the 4-week-old wild-type plants increased salt stress tolerance in terms of photosynthetic pigments contents under salt stress, while such positive effect of JA was insignificant in the *myc2* mutants (Yastreb et al., 2015). In addition, salt stress-repressed lateral root growth in the wild-type seedlings is enhanced in the *Glucosinolate Transporter1* (*GTR1*) mutant, which is characterized as a transporter of a bioactive form of JA, JA-Ile (Kuo et al., 2020), demonstrating that JA signaling functions in plant root growth and development during plant response to high salinity. Together with the previous studies, the roles of JA in plant salt stress tolerance are sophisticated and may be species- or conditions-dependent. Further experiments are still required to gain more insights into the mechanisms of JA underlying plant salt stress responses.

## Data Availability Statement

The raw data supporting the conclusions of this article will be made available by the authors, without undue reservation.

## Author Contributions

W-CL conceived the studies and designed the work. R-FS and T-TL performed most of the experiments and analyzed the data. W-CL wrote the manuscript with contribution and approval from all authors. All authors contributed to the article and approved the submitted version.

## Funding

This work was supported by the National Natural Science Foundation of China (#32000150), the Henan Young Talents Projects (#2020HYTP038), and the Program for Innovative Research Team (in Science and Technology) in University of Henan Province (21IRTSTHN019).

## Conflict of Interest

The authors declare that the research was conducted in the absence of any commercial or financial relationships that could be construed as a potential conflict of interest.

## Publisher's Note

All claims expressed in this article are solely those of the authors and do not necessarily represent those of their affiliated organizations, or those of the publisher, the editors and the reviewers. Any product that may be evaluated in this article, or claim that may be made by its manufacturer, is not guaranteed or endorsed by the publisher.
