# Supplementary material for: Jasmonic Acid Impairs Arabidopsis Seedling Salt Stress Tolerance Through MYC2-Mediated Repression of CAT2 Expression
Source: Front Plant Sci. 2021 Oct 22;12:730228. doi: 10.3389/fpls.2021.730228 (PMC8569249; doi:10.3389/fpls.2021.730228)
Supplement: Supplementary file 1 [file Table_1.docx]

**Supplemental Table S1**

List of the primers used in this study.

**Primer name Sequence (5’ to 3’)**

**Primers used for qRT-PCR**

CAT2-qF tcaaaccatggatccttacaagt

CAT2-qR tgttccatacaggagcacca

ACTIN2/8-qF GGTAACATTGTGCTCAGTGGTGG

ACTIN2/8-qR AACGACCTTAATCTTCATGCTGC

ACX2-qF CGGATCCAACGATTGTCTTTAC

ACX2-qR GTCGGGAATTGAAAAAGTCGAA

ACX3-qF GATCACAATGAAACGGATCTGG

ACX3-qR AGACGGAGTGATCATAAATCCC

RBOHD-qF AGCTTCACAATTATTGCACGAG

RBOHD-qR TCTCCAGTTAGGTTTAGCGAAG

RBOHF-qF TATTGGAGACCATCTTGCTTGT

RBOHF-qR CGTTAAAACCGGTTAGTCGATC

GOX1-qF GAGATCACTAACGTTACCGAGT

GOX1-qR CTCTTGAAGAGTCCATTGGTCT
