# Supplementary material for: Jasmonic Acid Impairs Arabidopsis Seedling Salt Stress Tolerance Through MYC2-Mediated Repression of CAT2 Expression
Source: Front Plant Sci. 2021 Oct 22;12:730228. doi: 10.3389/fpls.2021.730228 (PMC8569249; doi:10.3389/fpls.2021.730228)
Supplement: Supplementary file 2 [file Table_2.DOCX]

**Supplemental Table 2. The table of two-way ANOVA results in Figure 1F.**

| **Two-way ANOVA table** | **SS** | **DF** | **MS** | **F (DFn, DFd)** | **P value** |
| --- | --- | --- | --- | --- | --- |
| Interaction | 1.236 | 1 | 1.236 | F (1, 36) = 20.79 | P<0.0001 |
| Row Factor | 31.67 | 1 | 31.67 | F (1, 36) = 532.9 | P<0.0001 |
| Column Factor | 6.36 | 1 | 6.36 | F (1, 36) = 107 | P<0.0001 |
| Residual | 2.139 | 36 | 0.05942 |  |  |
| Number of missing values | 0 |  |  |  |  |
