# Supplementary figures and images for: Jasmonic Acid Impairs Arabidopsis Seedling Salt Stress Tolerance Through MYC2-Mediated Repression of CAT2 Expression
Source: Front Plant Sci. 2021 Oct 22;12:730228. doi: 10.3389/fpls.2021.730228 (PMC8569249; doi:10.3389/fpls.2021.730228)

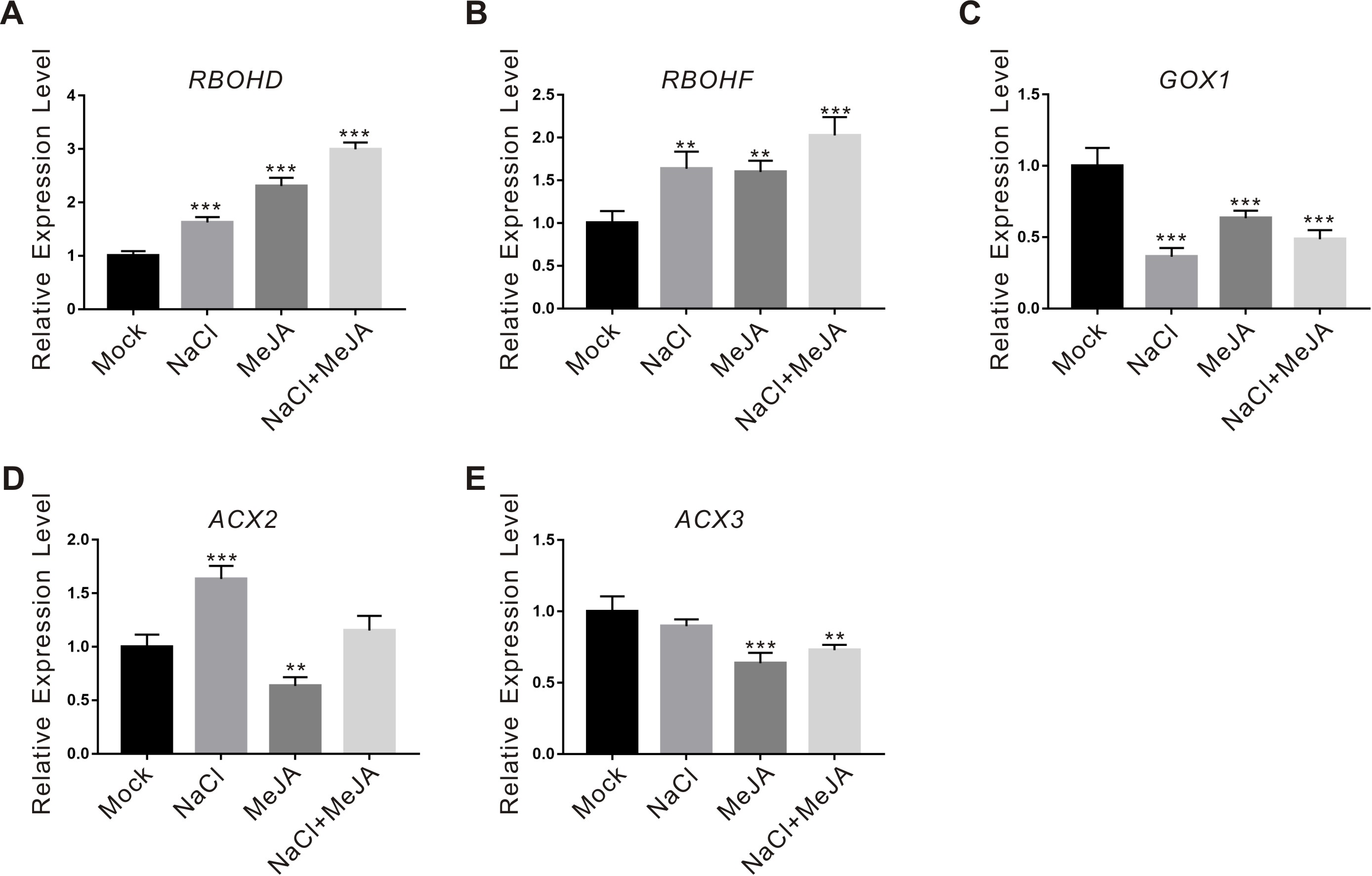

Supplement: Supplementary file 3 [file Image_1.JPEG]
